# A Polymorphism in the IL-5 Gene is Associated with Inhibitor Development in Severe Hemophilia A Patients

**DOI:** 10.4274/Tjh.2012.0197

**Published:** 2014-03-05

**Authors:** İnanç Değer Fidancı, Bülent Zülfikar, Kaan Kavaklı, M. Cem Ar, Yurdanur Kılınç, Zafer Başlar, Server Hande Çağlayan

**Affiliations:** 1 Boğaziçi University, Department of Molecular Biology and Genetics, İstanbul, Turkey; 2 İstanbul University Medical School, Institute of Oncology, İstanbul, Turkey; 3 Ege University Medical School, Department of Pediatric Hematology, İzmir, Turkey; 4 İstanbul Training and Education Hospital, Department of Hematology, İstanbul, Turkey; 5 Çukurova University Medical School, Department of Pediatric Hematology, Adana, Turkey; 6 İstanbul University Cerrahpaşa Medical Faculty, Department of Internal Medicine, Division of Hematology, İstanbul, Turkey

**Keywords:** Hemophilia A, Inhibitor formation, F8 gene mutation, Single nucleotide gene polymorphisms, Interleukins/cytokines, Association study

## Abstract

**Objective:** A severe complication in the replacement therapy of hemophilia A (HA) patients is the development of alloantibodies (inhibitors) against factor VIII, which neutralizes the substituted factor. The primary genetic risk factors influencing the development of inhibitors are F8 gene mutations. Interleukins and cytokines that are involved in the regulation of B-lymphocyte development are other possible targets as genetic risk factors. This study assesses the possible involvement of 9 selected single nucleotide gene polymorphisms (SNPs) with interleukins (IL-4, IL-5, and IL-10), transforming growth factor beta 1 (TGF-β1), and interferon gamma (IFN-γ) in inhibitor development in severely affected HA patients carrying a null mutation in the F8 gene.

**Materials and Methods:** A total of 173 HA patients were screened for intron 22 inversion and null mutations (nonsense and deletions). Genotyping of a total of 9 SNPs in genes IL-4, IL-5, IL-10, TGF-β1, and IFN-γ in 103 patients and 100 healthy individuals was carried out.

**Results:** An association analysis between 42 inhibitor (+) and 61 inhibitor (-) patients showed a significant association with the T allele of rs2069812 in the IL-5 gene promoter and patients with inhibitors (p=0.0251). The TT genotype was also significantly associated with this group with a p-value of 0.0082, odds ratio of about 7, and confidence interval of over 90%, suggesting that it is the recessive susceptibility allele and that the C allele is the dominant protective allele.

**Conclusion:** The lack of other variants in the IL-5 gene of patients and controls suggests that rs2069812 may be a regulatory SNP and may have a role in B-lymphocyte development, constituting a genetic risk factor in antibody development.

## Abstract (tr)

**Amaç: **Hemofili A hastalarının replasman tedavisinde FVIII’i nötralize eden FVIII antikorların (inhibitör) oluşması ciddi bir komplikasyondur. F8 mutasyonları ile birlikte başka genetik risk faktörleri de inhibitor gelişimini etkilemektedir. Bunlar arasında B-lenfositlerinin regülasyonunda yer alan IL-4, IL-5, IL-10, TGF-β1 ve IFN-γ gibi interlökin ve sitokinler diğer genetik risk faktörleri olabilecek hedeflerdir. Bu çalışmanın amacı inhibitor geliştiren ağır hemofili hastalarında çeşitli yöntemlerle F8 mutasyon profilini ortaya çıkarmak ve bunu takiben, FVIII yapılmaması ile sonuçlanan F8 mutasyonlu inhibitör geliştiren HA hastalarında 9 seçilmiş interlökin ve sitokin gen polimorfizmleri ile inhibitor gelişimi arasındaki ilişkiyi irdelemektir.

**Gereç ve Yöntemler:** Toplam 173 hasta intron 22 inversiyon mutasyonu ve null mutasyonlar (nonsense ve delesyon mutasyonları) için genetik anlamda taranmıstır. Daha sonra hasta (103) ve sağlıklı birey grupları (100) IL-4, IL-5, IL-10, TGF-β1 ve IFN-γ genlerinde bulunan 9 SNP bölgesi için araştırılmıştır.

**Bulgular:** İnhibitörlü hastalarda en sık rastlanan FVIII işlevini önemli ölçüde etkileyen mutasyonlar, sırasıyla, intron 22 inversiyonu, anlamsız mutasyon ve büyük delesyonlardır. Bu sebeple, bir hasta-kontrol ilişkisi çalışması şeklinde inhibitor (+) ve inhibitor (-) hasta altgrupları oluşturmak için ağır HA hastalarında intron 22 inversiyonu taranmıştır. IL-5 geni promotör bölgesinde yer alan rs2069812’nin T-aleli ile inhibitörlü hastalar arasında p-değeri 0,0251 olan önemli bir ilişki bulunmuştur. TT genotipinin de 0,0082 p-değeri, OR=7 ve %90 ustu CI ile inhibitör (+) grubu ile ilişkili olması T-alelinin çekinik yatkınlık aleli ve C-alelinin baskın koruyucu alel olduğunu düşündürmektedir.

**Sonuç:** Bu bulgular B lenfosit gelişiminde yer alan gen polimorfizmlerinin FVIII yapımı olmayan inhibitörlü ağır HA hastalarında oynadığı rol hakkında önemli bilgi vermekte ve bu alanda ileri çalışmalara önderlik etmektedir.

## INTRODUCTION

The major complication of replacement therapy is the development of antibodies (inhibitors), which inhibit factor VIII (FVIII) activity in hemophilia A (HA). Inhibitor formation occurs in 20%-30% of patients with severe HA. Both genetic and non-genetic factors play crucial roles in the development of inhibitors against FVIII protein [1]. Genetic factors including mutations or polymorphisms within the factor 8 (F8) gene, some immune response genes like major histocompatibility complex (MHC) class I/II, interleukins (ILs), and cytokines were shown to be decisive risk factors in inhibitor development [2]. However, the same type of F8 gene mutation can be seen in HA patients both with and without inhibitors. Patients with large deletions affecting more than one domain of the FVIII protein are at the highest risk of inhibitor development (75%). Nonsense mutations on the light chain increase the risk of inhibitor development much more than those on the heavy chain. The third highest risk mutation is the intron 22 inversion, with an inhibitor risk about 30%-35% [3]. We have previously reported that the most prevalent F8 gene mutation in severe HA patients with inhibitors is intron 22 inversion, with a frequency of 50% [4]. Risk of inhibitor development increases at times of severe bleeding, trauma, or surgery, especially when high doses of FVIII are used for treatment. This occurs as a result of complicated immune reactions leading to the up-regulation of T- and B-cell responses [5]. In the presence of foreign FVIII, CD4+ T-cells are induced to differentiate into T helper (Th1 and Th2) cells by secreting IL-12 and IL-18. Cytokines secreted by the Th1 [(IL-2 and interferon gamma (INF-γ)] and Th2 (IL-4, IL-5, and IL-10) cells direct B-cell synthesis for antibodies that function as inhibitors against FVIII. However, Th2 cells can also down-regulate B-cell antibody synthesis under certain circumstances [6]. A strong association with increased risk of inhibitor development and the presence of a 134-bp allele in one of the cytosine adenine (CA) repeat microsatellites (IL-10G) located in the promoter region of the IL-10 gene has been recently reported. IL-10 was the first gene located outside the causative F8 gene mutations shown to be linked to inhibitor development [7]. The single nucleotide polymorphism (SNP) in the promoter region of tumor necrosis factor alpha (TNF-α) was shown to be strongly associated with inhibitor formation in HA siblings in the Malmö International Brother Study [8]. A C/T SNP in the promoter region of the cytotoxic T-lymphocyte antigen-4 (CTLA-4) gene was found to be associated with inhibitor formation in 31.2% of T allele carriers (p=0.012) [9]. The aim of this study was to search for other genetic risk factors that may be associated with inhibitor development. For this purpose, informative SNPs of cytokine genes (IL-4, IL-5, IL-10, TGF-β1, and IFN-γ) involved in the regulation of B-cell responses were studied in a group of HA patients with null mutations (mutations with a major effect).

## MATERIALS AND METHODS

**Patients**

A total of 173 HA patients were screened for the presence of intron 22 inversion and other null mutations. Three patients had moderate phenotypes with FVIII activity of 2%-4%, while the remaining 170 patients had severe phenotypes with FVIII activity of 0%-3% [1]. One hundred and fourteen patients (66%) had no inhibitor history. Forty-two (24%) and 17 patients (10%) of the remaining 59 patients had high and low titer inhibitors, respectively. The median age was 22.6 years (range: 4-50 years). Individual phenotypic characteristics of the 173 unrelated HA patients are given as supplementary information. Intron 22 inversions were detected in 95 patients (54%), of whom 34 had inhibitors and 61 had no inhibitors. Three nonsense mutations and 5 deletions were detected as other types of null mutations, and all of these patients had developed inhibitors. Forty-two and 61 patients, therefore, constituted the 2 groups with and without inhibitors, respectively, for the association analysis (Table 1). 

The peripheral blood samples from 173 unrelated severe HA patients with and without inhibitors were collected from various hematology clinics within Turkey. The diagnosis of HA was based on clinical and hematological data. One-stage clotting assay was used for measurement of FVIII activity (Sigma Diagnostic, St. Louis, MO, USA). All measurements were performed in duplicate. The mean±standard deviation value for FVIII was 113.98±33.86 U/dL in control subjects. Values over 150 U/dL were accepted as high. The clinical criteria of Eyster et al. were used to determine disease severity [10]. The level of inhibitors was measured as Bethesda units (BU/mL). Patients with <5 BU/mL and >5 BU/mL were defined as having “low titer” and “high titer” inhibitors, respectively [6]. The study was approved by the Ethics Committee of Ege University Medical School. All included patients and healthy volunteers gave written consent before entering the study.

**DNA Extraction**

DNA was extracted from 10 mL of peripheral blood of patients by the NaCl method with 2 mL of peripheral blood using a MagNA Pure Compact instrument (Roche Diagnostics, Mannheim, Germany) and from saliva samples of some of the control individuals with the ORAGENE saliva kit (DNA Genotek, Kanata, ON, Canada). 

**Detection of Intron 22 Inversion**

Intron 22 inversion was detected by inverse polymerase chain reaction (PCR) [11] and long PCR techniques [12]. 

**Detection of F8 Nonsense and Deletion Mutations**

Intron 22 (-) patients were also screened for missense mutations with exon-specific PCR amplifications. All 26 exons of those patients were amplified and sequenced by automated Sanger sequencing.

**Selection of the SNPs in Immune Response Genes for Association Study**

Nine SNPs in genes IL-2, IFN-γ, IL-4, IL-5, IL-10, and TGF-β1 were selected with average heterozygote frequency close to 0.5 in different populations from HapMap and NCBI data.

Genotyping for the Case-Control Association Study

Genotyping of a total of 9 SNPs in 173 patients and 100 healthy individuals was carried out using hybridization probes designed by TIB MOLBIOL (Berlin, Germany) on an LC480 platform (Roche Diagnostics) based on melting curve analysis.

**DNA Sequence Analysis**

The IL-5 gene was divided into 7 regions for high-resolution melting analysis (HRM) and DNA sequencing. Five regions consisting of promoter and coding regions of the IL-5 gene were amplified in PCR reactions that contained 50 ng of genomic DNA, 0.2 pmol of each primer, 0.2 mM of each dNTP, 1X reaction buffer with 2 mM Mg2+, and 1.25 U Taq polymerase in 25 µL. The PCR products of these regions were directly sequenced (Macrogen, Seoul, Korea), whereas the remaining 2 regions were analyzed by HRM on an LC480 platform. The HRM mixture was prepared in a 20 µL volume containing 1X master mix with FastStart Taq DNA polymerase, reaction buffer, dNTP mix, and high-resolution melting dye; 0.2-0.5 mM Mg2+; 0.2-0.5 pmol of each primer pair; and 20-40 ng of genomic DNA. 

**Copy Number Variation Analysis by qPCR**

A quantitative PCR (qPCR) assay was used to detect copy number variations (CNVs) of the IL-5 gene rs2069812 region in patients. Absolute quantification using the “Fit Points Method” is an analysis used to quantify the target sequence and reference sequence and gives a concentration value. Real-time qPCRs were performed with a LightCycler 480 instrument and a LightCycler 480 SYBR Green I Master Kit and target and reference sequence-specific primers. The target sequence was the IL-5 rs2069812 region and the reference sequence was exon 6 of the sodium channel 1 alpha (SCN1A) gene (Ex6F-5’ CACACGTGTTAAGT, Ex6R-5’ AGCCCTCAAGTAT).

**Statistical Analysis **

Case-control association analysis was carried out with Haploview 4.1, which calculated the chi-square statistics of SNP alleles between 2 groups at a 0.05 significance level. Genotype frequencies were calculated in patients for 9 SNPs by using the chi-square test on a webpage of the University of Kansas (http://people.ku.edu/~preacher/chisq/chisq.htm). Multiple test correction was done by 100.000 permutations (p=0.0294). Other association tests were performed by using crude, recessive, or dominant models [13]. Power analysis was carried out by assuming an inhibitor development rate of 0.00018 in the general population and a type I error rate of 0.05. The power was over 90% for a recessive genotype effect with a relative risk (RR) of 6.86 for rs2069812. All power calculations were carried out by QUANTO [14,15].

## RESULTS

**SNP Genotyping in Immune Response Genes**

Genotyping of 1 SNP failed for healthy controls, but 8 SNPs were genotyped in 100 healthy Turkish individuals by HybProbe probes. All SNPs were in Hardy-Weinberg equilibrium and each had a minor allele frequency of >0.120. Genotyping of 9 SNPs in 42 inhibitor (+) and 61 inhibitor (-) severe HA patients revealed that they were in Hardy-Weinberg equilibrium in both patient groups. They had a minor allele frequency of higher than 0.19 and 0.13 for the inhibitor (-) and inhibitor (+) patient groups, respectively. These 2 subgroups, both with known F8 null mutations, comprised the cases and controls, and the association analysis was carried out using Haploview 4.1. The associated alleles and p-values are given in Table 2. The T allele of rs2069812, which resides in the IL-5 gene promoter region, was found to be associated with inhibitor (+) patients with a p-value of 0.0251. Multiple test correction was done with 100.000 permutations (p=0.0294). The test for association was repeated using all patients [i.e. inhibitor (+) and inhibitor (-) groups] against healthy individuals and no significant associations were detected, supporting the association of rs2069812 with inhibitor formation. Genotype frequencies were calculated in 2 patient groups for 9 SNPs by using the chi-square test a webpage of the University of Kansas (http://people.ku.edu/~preacher/chisq/chisq.htm). The TT genotype of rs2069812 was found to be associated with inhibitor (+) patients with a p-value of 0.0082 (Table 3). These results were compatible with the results of the Haploview 4.1 case-control association analysis. A similar association test run between inhibitor (+) patients and healthy individuals did not reveal any significant associations. The pattern of inheritance of rs2069812 indicated a similar and reduced risk for CT and CC genotypes in inhibitor (+) patients in the crude genetic model (Table 4) [13]. In the model where the T allele was recessive, the TT genotype carried a risk of 6.86-fold compared to CT or CC genotypes, indicating that the T allele was the susceptibility allele. On the other hand, considering that the C allele has a dominant inheritance, CT or CC genotypes reduced the disease risk by 0.02% (odds ratio=0.14). Therefore, the C allele could be considered to have a dominant protective effect. 

**HRM and DNA Sequence Analysis of the IL-5 Gene**

The IL-5 gene is composed of 4 exons spanning a 2078-bp coding region. In order to detect any pathological changes segregating with the associated rs2069812, the IL-5 gene was divided into 7 regions for HRM and direct DNA sequencing. The promoter region containing rs2069812 was divided into 3 regions. Two of the promoter regions and exons 1, 2, and 4 were amplified by PCR and sequenced in 103 patients. The promoter 1 region and exon 3 of the IL-5 gene were analyzed by HRM in real time in the same patients. Sequence analysis revealed the genotypes of 14 other known SNPs located in the IL-5 gene, but no other rare variants. There were no haplotype associations between these SNPs when tested by the Haploview program. 

**CNV Analysis of rs2069812 Region**

In order to investigate the presence of CNVs, real-time qPCR analysis was applied to patients who had homozygote and heterozygote genotypes for associated SNP region rs2069812. Absolute quantification analysis was used to quantify the target sequence and reference sequences. Relative quantification was used to compare these targets and reference sequence concentrations. The target sequence was the IL-5 promoter, including the rs2069812 region, and the reference sequence was exon 6 of the SCN1A gene. qPCR assay was performed for 28 homozygous (for rs2069812) inhibitor (+) patients and 30 homozygous inhibitor (-) patients in 2 groups. The ratio of the normalized target sequence to the reference sequence was near 1, which meant that there were no copy number changes in this region. qPCR assay was also performed for 14 heterozygous (for rs2069812) inhibitor (+) patients and 31 heterozygous inhibitor (-) patients in 2 groups. The ratio of the normalized target sequence to the reference sequence was also near 1 (data not shown).

## DISCUSSION

Genetic variants including SNPs, CNVs, or mutations in immune response genes other than F8 gene mutations may affect inhibitor development in patients with severe HA and cause major complications. It has been proposed that immune response can be up-regulated in most patients with null mutations like intron 22 inversion [3]. In studies of patients with autoimmune disease, polymorphisms in the immune response genes have been found to be associated with antibody formation [21]. In HA patients certain alleles in the promoter regions of the IL-10, TNF-α, and CTLA-4 genes were found to be associated with inhibitor development [7,8,9]. 

In this study, in order to further understand the role of immune response genes on inhibitor development, patients with a null F8 gene mutation with high prevalence, like intron 22 inversion, were grouped as patients with and without inhibitors and an association analysis was carried out between them using 9 SNPs located in the IL-2, IFN-γ, IL-4, IL-5, IL-10, and TGF-β1 genes. A significant association was seen with the T allele of rs2069812 in the IL-5 promoter and inhibitor positivity in patients with HA. The analysis for the inheritance pattern revealed that carrying the TT genotype for rs2069812 meant a 6.86 times greater probability of developing inhibitors. On the other hand, patients carrying CT or CC had that risk at a rate of 0.02% (OR=0.14) as compared to the TT genotype. Therefore, the T allele was considered as a recessive susceptibility allele and the C allele as a dominant protective allele. The resulting association of the T allele of rs72069812 with over 90% power for a recessive genotype effect indicated a possible role in inhibitor development in inhibitor (+) patients. DNA sequencing analysis of the IL-5 gene promoter and coding sequences and qPCR analysis of the region involving the associated SNP in 103 patients did not reveal any common/rare variants segregating with the associated SNP.

IL-5 is an immune response gene whose product plays a role in B-cell antibody synthesis. The IL-5 gene expresses the IL-5 glycoprotein, which plays a pleiotropic role in the immune system and inflammation. It supports growth and differentiation of B cells and has a key mediator role in eosinophil activation. It is produced by Th2 cells and masT-cells. IL-5 cytokines are the key molecules in allergy and eosinophilic inflammation [16]. In previous studies, rs2069812 was found to be associated with diseases like atopic bronchial asthma [17], gastric cancer risk [18], and atopic dermatitis [19]. The IL-5 gene is expressed in CD4+ T-cells, masT-cells, and eosinophils, and in allergic reactions the expression level of the IL-5 gene can be varied [16]. It may be suggested that rs2069812 localized in the gene promoter could be a regulatory SNP and play a role in the up-regulation or down-regulation of the IL-5 gene and influence the level of IL-5 protein. It could further be suggested that the T variant in inhibitor (+) patients causes increased or decreased production of IL-5 protein, leading to inhibitor formation. In order to see the specific IL-5 gene expression against exogenous FVIII protein, CD4+ T-cells with the T and C alleles of rs72069812 responding to FVIII antigens need to be isolated from peripheral blood and treated with FVIII protein in cell culture studies. In silico analysis, however, does not reveal any alteration in the transcription factor binding scores of either the T or the C allele of rs2069812 (http://alggen.lsi.upc.es/cgi-bin/promo_v3/). 

In addition, SNPs may be involved in epigenetic regulation since some SNPs and CpG sites show significant cis- or trans-associations. It was hypothesized that a considerable proportion of CpG sites may be quantitative traits with regard to regulation by specific genetic variants [20]. rs2069812 is not located at a CpG site and a CpG island was not detected within approximately 5000 bases of the 5’ region of the IL-5 gene (http://www.ualberta.ca/~stothard/javascript/cpg_islands.html), suggesting that it is not a cis-regulatory SNP. However, the IL-5 gene, together with genes IL-4, IL-13, and colony stimulating factor 2 (CSF-2), form a cytokine gene cluster on chromosome 5q31. CSF-2, IL-4, and IL-13 are regulated coordinately by long-range regulatory elements of 120 kb in length on chromosome 5q31. When this region was scanned for CpG islands, approximately 70 CpG islands were found. Whether rs2069812 is in cis- or trans-association with a distant CpG island remains to be studied further.

In conclusion, the associated T allele of the promoter SNP in the IL-5 gene may be part of the complex genetic background involved in the development of inhibitors in severe HA patients.

## ACKNOWLEDGMENTS

We thank Dr. Tiraje Celkan, Department of Pediatrics, Cerrahpaşa Medical School, İstanbul University, İstanbul, Turkey; Dr. Çetin Timur, Department of Pediatrics and Hematology, Göztepe State Hospital, İstanbul, Turkey; Dr. Canan Vergin, Dr. Behçet Uz Pediatric Hospital, İzmir, Turkey; and Dr. Canan Uçar, Department of Pediatric Hematology, 19 Mayıs University Medical School, Samsun, Turkey, for providing patient samples. We greatly appreciate the help of Dr. Fikret Bezgal from the Turkish Hemophilia Society and the technical help of Aslı Gündoğdu, and we are grateful to all patients for their participation in this study. This study was financially supported by TÜBİTAK (the Scientific and Technological Research Council of Turkey) project number 108S095; Boğaziçi University Research Foundation projects 09HB101D, 08HB104D, and 07HB103D; and Eczacıbaşı-Baxter, İstanbul, Turkey. 

İnanç D. Fidancı designed the study, performed the experiments, performed the analysis, and wrote the manuscript. Bülent Zülfikar, Kaan Kavaklı, Yurdanur Kılınç, and Cem Ar provided the patient samples and clinical information. Zafer Başlar followed up on the inhibitor development in most of the patients. S. Hande Çağlayan designed the study, performed the analysis, and wrote the manuscript.

None of the authors have any conflict of interest to disclose 

We confirm that we have read the journal’s position on issues involved in ethical publication and affirm that this report is consistent with those guidelines.

## CONFLICT OF INTEREST STATEMENT

The authors of this paper have no conflicts of interest, including specific financial interests, relationships, and/ or affiliations relevant to the subject matter or materials included.

## Figures and Tables

**Table 1:**
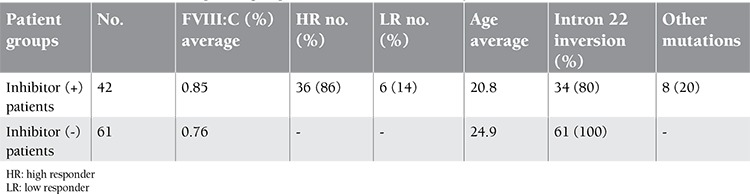
Clinical information of patient groups used in the association analysis

**Table 2:**
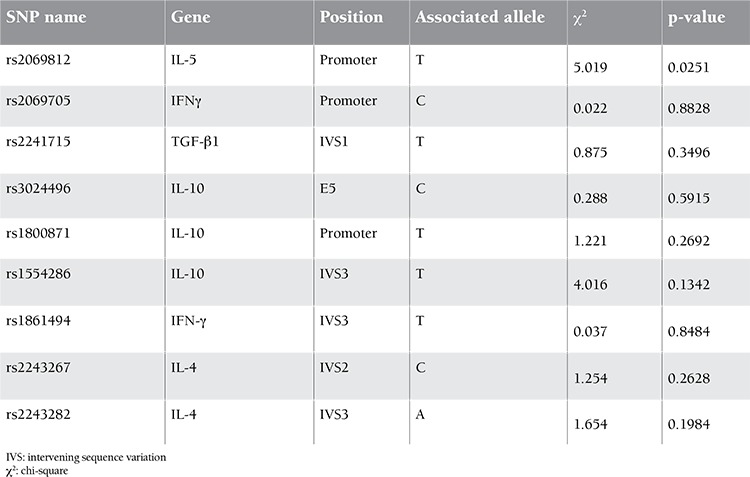
Test of association between inhibitor (+) and inhibitor (–) patient subgroups

**Table 3:**
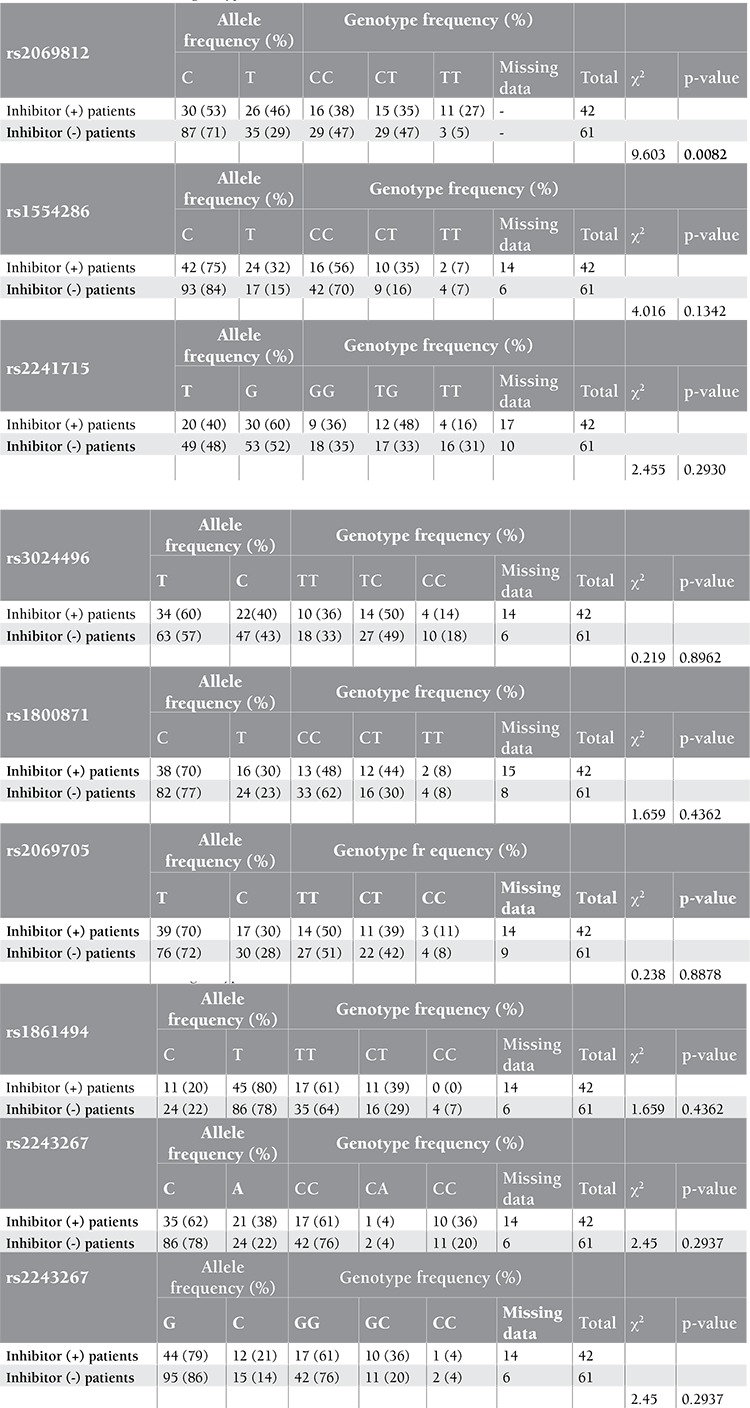
Test of association of genotypes of 9 SNPs

**Table 4:**
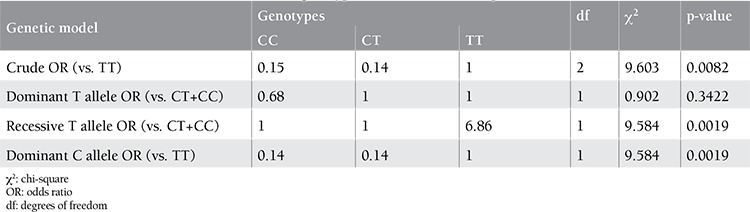
Test of association between rs2069812 genotypes and inhibitor development
